# Changes in the Expression of MIF and Other Key Enzymes of Energy Metabolism in the Myocardia of Broiler Chickens with Ascites Syndrome

**DOI:** 10.3390/ani12192488

**Published:** 2022-09-20

**Authors:** Lifang Li, Qiufeng Jia, Lingli Chen, Wenkui Wang

**Affiliations:** 1 College of Veterinary Medicine, Shanxi Agricultural University, Jinzhong 030801, China; 2 Department of Mathematics and Physics, Jinzhong College of Information, Jinzhong 030800, China; 3 College of Animal Science and Veterinary Medicine, Henan Institute of Science and Technology, Xinxiang 453003, China

**Keywords:** broiler, ascites syndrome, macrophage migration inhibitory factor, AMP-activated protein kinase, glucose metabolism, fatty acid metabolism

## Abstract

**Simple Summary:**

Ascites syndrome (AS), a nutritional, metabolic disease in broiler chickens, exerts serious effects on the economic efficiency of the broiler industry. To date, the pathogenesis of broiler ascites syndrome is still not well understood. In this study, we explored the metabolic function of the right ventricles of clinical ascitic broilers and healthy broilers (control) from the same flock. Our study showed the myocardial energy supply in ascitic broiler chickens occurs mainly through glycolysis and fatty acid metabolism, and the oxidation of the TCA cycle is blunted. These findings suggest that there is insufficient energy metabolism in the right hearts of broilers with ascites syndrome, causing a state of functional failure. These results may provide useful information for elucidating the pathogenesis of broiler ascites syndrome and may also provide a reference for future research on similar diseases (including pulmonary hypertension syndrome and heart failure) in humans and domestic animals.

**Abstract:**

Ascites syndrome (AS) is a metabolic disease observed mainly in fast-growing broilers. The heart is one of the most important target organs of the disease. The goal of this study was to evaluate the metabolic function of the right ventricles in clinical ascitic broilers. HE staining was performed to observe histopathological changes in the right ventricle of the heart, while Western blotting was used to detect the protein expression levels of macrophage migration inhibitory factor (MIF) and phosphorylated AMP-activated protein kinase (p-AMPK), as well as other key enzymes of energy metabolic pathways (i.e., glycolytic pathway: HK2, PFK1, PFK2, and PKM2; the tricarboxylic acid cycle (TCA cycle) pathway: OGDH, IDH2, and CS; and the fatty acid oxidation pathway: CPT-1A and ACC) in myocardial tissue. The histopathological examination of the myocardia of ascitic broilers revealed disoriented myocardial cells in the myofibril structure and a large number of blood cells deposited in the intermyofibrillar vessels, suggesting right heart failure in ascitic broilers. The Western blotting analysis demonstrated significantly increased levels of MIF and p-AMPK in the myocardia of ascitic broilers compared to those of the control group (*p* < 0.05). Additionally, the protein expression of key enzymes was dramatically increased in the glycolytic and fatty acid oxidation pathways, while the protein expression of key enzymes in the TCA cycle pathway was decreased in the ascitic broiler group. These findings suggest enhanced glycolysis and fatty acid oxidation metabolism, and a diminished TCA cycle, in the myocardia of broiler chickens with ascites syndrome.

## 1. Introduction

Ascites syndrome (AS), also known as pulmonary hypertension syndrome (PHS), is a nutritional, metabolic disease in broiler chickens. It is characterized by abdominal fluid accumulation and right ventricular hypertrophy [1,2,3]. This disease exerts serious effects on the economic efficiency of the broiler industry and the quality of chicken meat [4,5], and has negative effects on animal welfare [6]. Many factors, such as physiological, environmental and management factors can cause this disease [7,8], which can be summarized as follows:

(1) Genetic factors: Broilers are artificially bred strains whose most significant biological characteristics are their exceedingly rapid growth rate and efficient feed conversion. However, they also possess genetic defects; for instance, an imbalance between the development of their cardio-respiratory system and their fast-growing bodies occurs before four weeks of age [9,10], which leaves their organs and muscles extremely vulnerable to hypoxia. Therefore, broiler chickens are especially prone to developing ascites.
(2) Triggering factors: Various stimuli that cause hypoxia may also induce the occurrence of ascites in broiler chickens. These stimuli include cold weather stress, light [11], high-energy diets, excessive sodium intake [12], and high altitudes [13,14,15].

To date, studies on the pathogenesis of ascites syndrome in broiler chickens has mainly focused on changes in the structure and functioning of the pulmonary arteries [2,9,16,17]. Long-term chronic hypoxia leads to the remodeling of the pulmonary artery [18] and pulmonary hypertension (PH), which can either result in right heart hypertrophy or the obstruction of the portal vein, both of which eventually lead to ascites. Although this mechanism is widely accepted, it does not fully reveal the pathogenesis of broiler ascites. This is because feeding restriction is still considered the simplest and most effective measure to prevent broiler ascites in real farming production [19,20,21]. This assumes that broiler ascites syndrome is a nutritional, metabolic disease, which requires the elucidation of the nature of the disease from the perspective of metabolic research. Moreover, previous studies on the heart are limited compared to those on the lungs [22,23], while the heart is one of the important target organs in this syndrome. Based on the above considerations, we hypothesized that cardiac energy metabolism may be altered in broiler chickens with ascites syndrome. We focused on cardiac energy metabolism in clinical cases with ascites syndrome from the perspective of energy metabolism.

## 2. Materials and Methods

### 2.1. Source and Screening of Ascitic Cases

A total of forty clinical ascitic broilers were obtained from AA broilers of poultry farms in the district of Taigu from the Da Xiang Group, Shanxi Province, China. The broilers were 25~42 days old, and their weight ranged from approximately 0.5 to 1.0 kg.

Suspected cases of ascites syndrome were selected and brought back to the laboratory. These chickens showed the following clinical signs: low mood, fluffed-out feathers, significantly enlarged abdomen, thinning skin (in some cases, fluid was even observed in the peritoneal cavity), and an obvious fluctuating sensation when pressed, but no other abnormal conditions [24]. The cases were first anesthetized using an intraperitoneal injection of urethane (1.5 g/kg of body weight) and then euthanized by vein bloodletting. Broilers were ultimately considered ascitic cases upon postmortem examination when the ratio of the weight of the right ventricle-to-total ventricle was not less than 0.30 (ascites heart index, AHI) [25]. We selected the same number of healthy broilers from the same flock to form the control group every time we picked suspected cases. The control and ascitic groups each included at least three replicates. The healthy chickens were treated identically.

The hearts of broilers were harvested, and the heart ventricles were cut out and weighed. Then, the right heart ventricles were cut out, weighed, and washed with sterilized saline. Next, one portion of the sample was stored at −80 °C, while the other was fixed in Bouin solution. All experiments involving the broilers were formally approved by the Institutional Animal Care and Use Committee of Shanxi Agricultural University, Taigu branch on 13 March 2018.

### 2.2. Antibodies and Chemicals

The phosphofructokinase 1 (PFK1) (sc-31711) antibody was purchased from Santa Cruz Biotechnology (Santa Cruz, CA, USA), while the phosphofructokinase 2 (PFK2) (bs-3528R), carnitine palmitoyltransferase-1A (CPT-1A) (bs-2047R), AMP-activated protein kinase (AMPK) (bs-1115R), β-actin (bs-10966R), phosphorylated PFK2 at Ser467 (bs-3331R), and phosphorylated pyruvate kinase M2 (p-PKM2) at Tyr105 (bs-3334R) antibodies were all purchased from Bioss Biotechnology (Beijing, China). The antibodies against PKM2 (15822–1-AP), hexokinase 2 (HK2) (22029–1-AP), α-ketoglutarate dehydrogenase (OGDH) (15212–1-AP), citrate synthase (CS) (16131–1-AP), isocitrate dehydrogenase 2 (IDH2) (15932–1-AP), lactate dehydrogenase B (LDHB) (14824–1-AP), and pyruvate dehydrogenase E1(PDHE1) (18068–1-AP) were all purchased from Proteintech Group (Chicago, IL, USA). The phosphorylated AMPK (Tyr172, 2531) antibody was acquired from Cell Signaling Technology (Beverly, MA, USA). The macrophage migration inhibitory factor (MIF) antibody was produced in our laboratory. Hematoxylin (AR11180–1) and eosin (AR11180–2) staining solutions were purchased from Boster Bioengineering Co. Ltd. (Wuhan, China), while the RIPA protein buffer and BCA protein assay kit (P0010) were procured from Beyotime Biotechnology (Shanghai, China). ECL-plus reagent (AR1111) was obtained from Bioss Biotechnology.

### 2.3. Hematoxylin and Eosin (HE) Staining

Myocardial tissues were fixed in Bouin solution for 48 h and then rinsed in running water for 24 h. Next, the tissues were dehydrated in an increasing alcohol gradient and cleared in xylene to remove residual alcohol. The tissues were ultimately embedded in paraffin, sectioned at a thickness of 4 µm, incubated in a water bath at 45~47 °C, and baked at 50 °C for 6~7 h. Finally, sections were stained with hematoxylin and eosin and observed under a light microscope.

### 2.4. Western Blotting

Western blotting was performed to detect the expression levels of proteins. Frozen myocardial tissue (100 mg) was powdered in liquid nitrogen and lysed in ice-cold RIPA buffer. The lysates were centrifuged at 4 °C and the supernatant (protein sample) was collected and stored for further use. The concentration of protein samples was determined using a BCA protein assay kit, and the proteins were separated according to their molecular weight using appropriate concentrations of sodium dodecyl sulfate-polyacrylamide gel (SDS-PAGE). Next, the proteins were transferred onto nitrocellulose membranes on an ice bath and blocked using 5% non-fat milk at room temperature for 2 h. Samples were incubated with primary antibodies at 4 °C overnight, followed by incubation with the corresponding secondary antibodies at room temperature for 2 h. The dilutions of primary antibodies are listed as follows: MIF (1:500), AMPK (1:500), p-AMPK (1:750), HK2 (1:2000), PFK1 (1:150), PFK2 (1:500), p-PFK2 (1:500), PKM2 (1:1000), p-PKM2 (1:300), OGDH (1:3000), CS (1:3000), IDH2 (1:1000), ACC (1:500), p-ACC (1:500), CPT-1A (1:300), PDHE1 (1:4000), LDHB (1:1000), β-actin (1:4000), and GAPDH (1:3000). The goat anti-mouse and goat anti-rabbit secondary antibodies were used at a dilution of 1:5000 and 1:4000, respectively. Finally, the protein bands were observed using an ECL-plus reagent.

### 2.5. Statistical Analysis

The target protein bands of Western blotting were detected using ImageJ (National Institutes of Health, Bethesda, MD, USA) to analyze the gray values. Data from at least three independent experiments are presented as the means ± SEM. Statistical analyses were performed using paired-sample *t*-tests with SPSS 19.0 (IBM Corp, Armonk, NY, USA). *p* < 0.05 is indicated by *; *p* < 0.01 is indicated by **.

## 3. Results

### 3.1. The Performance of Clinical Ascitic Broilers

**Figure 1:**
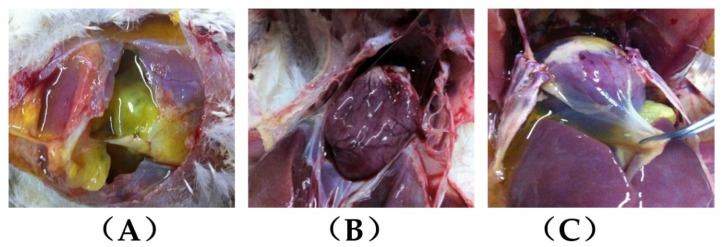
Autopsy results of AS broilers: (**A**) accumulation of large amounts of yellowish and clear fluid in the abdominal cavity without any fibrous clots; (**B**) the right ventricle shows flaccidity and poor elasticity; (**C**) pericardial effusion was observed in the heart.

The clinical observations and a preliminary judgment were followed by the postmortem of the clinical ascitic broilers, which revealed the following features: the accumulation of large amounts of yellowish fluid in the abdominal cavity without any fibrous clots (Figure 1A), 30 to 200 mL ascites, a significantly enlarged right ventricle showing a flabby and poorly elastic myocardium (Figure 1B), and pericardial effusion observed in the heart (Figure 1C). Meanwhile, the AHI in ascitic broilers was 0.303 ± 0.050, which was significantly higher (*p* < 0.01) than that of the control group (0.213 ± 0.024).

### 3.2. Myocardial Histopathology

**Figure 2:**
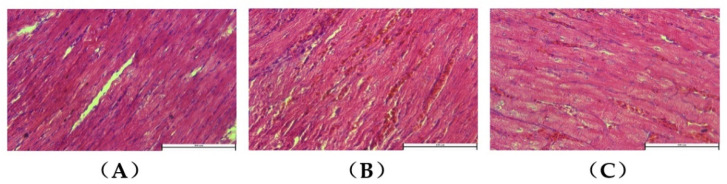
HE of broiler myocardial tissues (40×): (**A**) control group; (**B**,**C**) ascitic group, showing that, in some myocardial cells, the structure of myofibrils was disordered, and a large number of blood cells were deposited in the blood vessels.

HE staining of the control group showed neatly arranged myocardial fibers and structurally dense myofibrils, with no blood cells being deposited in the blood vessels between the myofibrils (Figure 2A). Compared to the control group, the ascitic group showed a neat arrangement of myofibrils. However, some cardiomyocytes exhibited disoriented myofibrils, with a large number of blood cells being deposited in the blood vessels between the myofibrils. This suggested the presence of myocardial injury and right heart ventricular dysfunction (Figure 2B,C).

### 3.3. Expressions of MIF and p-AMPK in Ascitic Broilers

**Figure 3:**
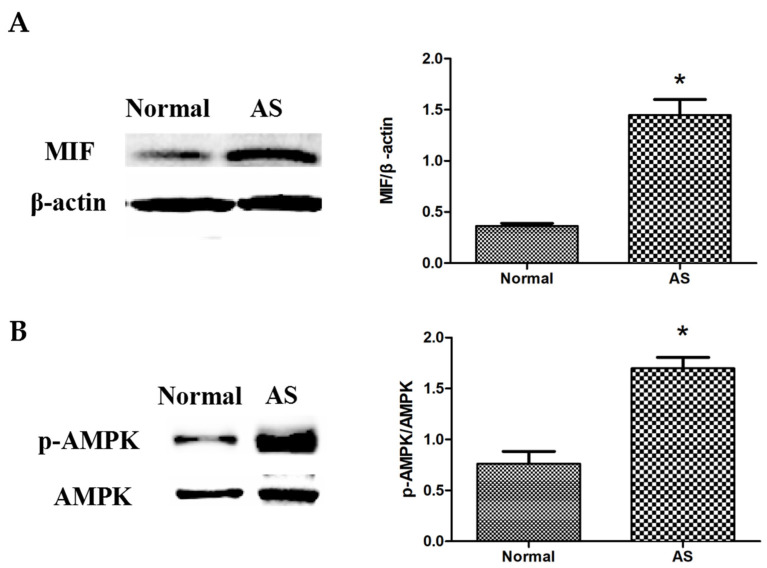
The protein expressions of MIF and p-AMPK in the control group and AS group were measured using Western blotting: (**A**) MIF; (**B**) p-AMPK and AMPK. The data are presented as the means ± SEM. The experiment was repeated three times independently. * *p* < 0.05 indicates a significant difference from the control group.

Western blotting showed a significantly upregulated expression level of MIF (Figure 3A) and p-AMPK (Figure 3B) in the myocardial tissues of ascitic broilers compared to that of the control broilers of the same flock (*p* < 0.05).

### 3.4. Expressions of Key Enzymes of the Energy Metabolic Pathway of Myocardial Tissue in Ascitic Broilers

**Figure 4:**
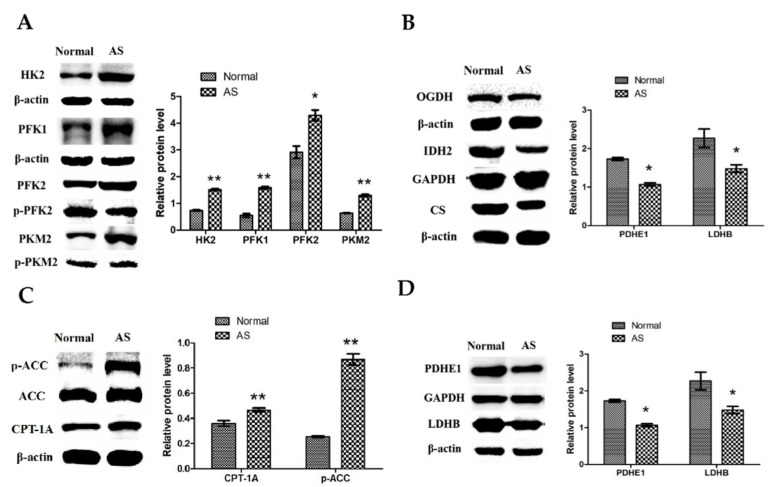
The protein expressions of key enzymes of the energy metabolic pathway of the myocardial tissues in ascitic broilers were measured using Western blotting: (**A**) the key enzymes PKM2, HK2, PFK1, and PFK2 in the glycolytic pathway of the ascitic broilers’ myocardial tissue; (**B**) the key enzymes OGDH, IDH2, and CS in the TCA cycle of the ascitic broilers’ myocardial tissue; (**C**) the key enzymes CPT-1A and p-ACC of the fatty acid oxidation pathway of the ascitic broilers’ myocardial tissue; (**D**) two important enzymes, PDHE1 and LDHB, of the glucose metabolism of the ascitic broilers’ myocardial tissue. The data are presented as means ± SEM. The experiment was repeated three times independently. * *p* < 0.05 and ** *p* < 0.01 indicate a significant difference from the control group.

Western blotting showed significantly enhanced activities of the key enzymes PKM2, HK2, PFK1, and PFK2 in the glycolytic pathway of the ascitic broilers’ myocardial tissue compared to those of the control broilers of the same flock (*p* < 0.05) (Figure 4A). This suggested that ascites in broilers led to enhanced energy supply in cardiac glycolysis.

As shown in Figure 4B, the expression levels of the key enzymes of the TCA cycle, OGDH, IDH2, and CS, were significantly decreased in the myocardial tissues of ascitic broilers compared to those of the control broilers in the same flock, indicating diminished aerobic oxidative energy supply to the heart in ascitic broilers.

As shown in Figure 4C, the expression levels of key enzymes of the fatty acid oxidation pathway, CPT-1A and p-ACC, were significantly higher in the myocardial tissues of ascitic broilers compared to those of the control broilers (*p* < 0.01). These results indicated an enhanced energy supply of the cardiac fatty acid oxidation process in ascitic broilers.

Additionally, the expression levels of both PDHE1 and LDHB were significantly decreased in the myocardial tissue of ascitic broilers compared to those of the control broilers (Figure 4D, *p* < 0.05). The weakened expression of LDHB suggested that pyruvate produced by the glycolytic pathway was mainly converted into lactate, which enhanced anaerobic oxidation. Meanwhile, the decreased expression of PDHE1 weakened the acetyl-CoA production entering the TCA cycle from the pyruvate of glycolysis origin, weakening the aerobic oxidation of glucose.

## 4. Discussion

Currently, ascites syndrome is still a clinically challenging disease: it has a detrimental impact on both humans and broilers [2]. Broiler ascites syndrome remains an important disease that cannot be ignored in broiler farming production due to its increasing incidence and mortality [26,27]. As we know, ascites syndrome is a nutritional, metabolic disease in broiler chickens, and the heart is among its important target organs; our study evaluated the cardiac energy metabolic status of clinical ascitic broilers. Conducting this research may not only improve the prevention and control of the disease, but can also improve the standard of broiler breeding, and may provide a reference for future research on similar diseases (including pulmonary hypertension syndrome and heart failure) in humans and domestic animals.

The clinically suspected cases of broiler ascites syndrome were screened based on the birds’ characteristics, including depression, fluffed-out feathers, and markedly enlarged and fluctuating abdomens [1,2,28]. An observed difference of this disease from other infectious diseases was that the body temperature of the birds was normal [29]. The results of pathological autopsy were characterized by the presence of a certain amount (more than 20 mL) of yellowish, clear, and transparent fluid in the peritoneal cavity as well as the hypertrophied right ventricle [25], with an AHI value of not less than 0.3. AHI is considered the gold-standard indicator for the diagnosis of broiler ascites syndrome. In the present study, combined with histopathological findings and the AHI result for the right ventricle in ascitic broilers, damaged myocardial structures were observed, suggesting right heart failure. These observations were similar to those of other researchers [7,30]. In addition, we also found some “suspected” cases, including those with a low volume of ascites (less than 20 mL), an enlarged right ventricle, and an AHI of less than 0.3, which were likely to be in a “transitional” period of disease onset and progression. Although they were of important research value, we excluded such “suspected” cases due to their atypical nature. However, for a more in-depth study in the future, we may also need to focus our attention on this aspect.

The heart beats every moment and is an extremely important organ that consumes a great amount of energy in both resting and exercising states. It is also one of the important target organs in broiler ascites syndrome, where hypoxia acts as the primary cause of its formation. Therefore, studying changes in energy metabolism in the heart of ascitic broilers is of great importance since it reveals the mechanism of pathogenesis of this syndrome. In eukaryotes, AMPK is considered a highly conserved “energy level sensor”. It is activated in energy deficit cases and inhibited in the case of energy excess conditions [31,32]. It is known as the “master switch” or “barometer” of energy metabolism, which protects the cells from “energy crisis”. Combining the results of our study with those of other researchers, right heart failure is an important feature of broiler ascites syndrome, and the cardiac energy metabolism may change accordingly. MIF is considered to be a vital link between these conditions. Many studies on mammalian cells have shown that MIF is a multifunctional cytokine with hormonal properties. It is highly expressed in cardiac myocytes and is closely related to hypoxia [33,34], with an important role in the regulation of myocardial energy metabolism [35]. Myocardium MIF is reported to improve myocardial energy metabolism by activating intracellular AMPK via acting on its membrane receptors [35,36], which in turn protects the heart from injury. Accordingly, we hypothesized that MIF may also play an important role in the regulation of energy metabolism and functional activities in the hearts of broilers. Therefore, in this study, we investigated the protein expression levels of both MIF and p-AMPK in ascitic broiler hearts.

**Figure 5:**
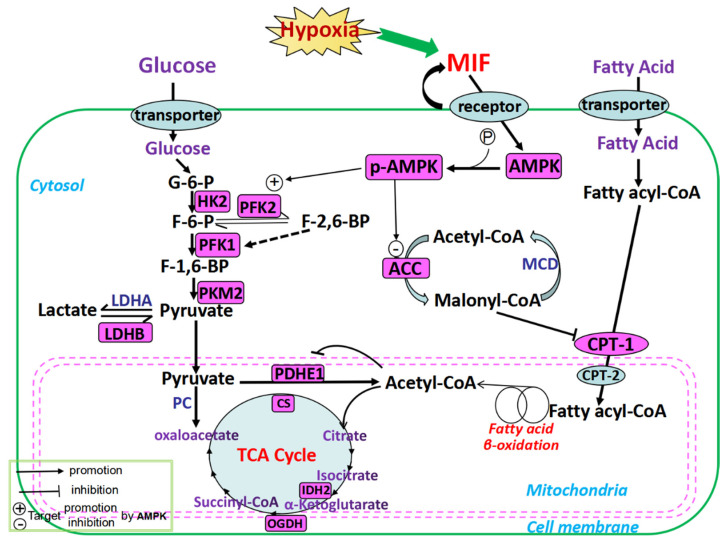
Schematic diagram of the mechanism of the key enzymes of myocardial energy metabolism.

Our results showed that, compared to the control group, the levels of MIF and p-AMPK were significantly increased in the myocardial tissues of ascitic broilers, suggesting a state of relative energy deficiency in the myocardia of ascitic broilers, where the feedback mechanism increased MIF secretion and elevated p-AMPK levels (Figure 5). This was consistent with the findings of other mammalian studies [36]. Activated AMPK produces more ATP through the regulation of key enzymes of glucose metabolism and fatty acid metabolism. Therefore, to investigate the effects of MIF and AMPK on the myocardial energy metabolism in ascitic broilers, we examined the key enzyme activities in glucose and fatty acid metabolic pathways in their myocardial tissues.

In the glucose metabolism pathway, the results showed that the activities of key enzymes of the glycolytic pathway, HK2, PFK1, PFK2, and PKM2, were significantly increased in ascitic broiler myocardial tissues compared to those of the control group, whereas the expression of the key enzymes of the TCA cycle, OGDH, IDH2, and CS, showed a significant decrease. These results indicated enhanced anaerobic glycolysis and reduced oxidative phosphorylation in the TCA cycle of myocardial tissues in the ascitic broilers. Therefore, under hypoxic conditions, the body adapts to the above-mentioned mechanism to produce more ATP when there are limited oxygen supply levels.

The expression levels of the key enzymes in the fatty acid oxidation pathway, CPT-1A and p-ACC, were significantly increased in the myocardia of the ascitic broilers compared to those of the control broilers. This suggested that the energy supply in the myocardia of the ascitic broilers was biased toward fatty acid oxidation. However, the occurrence of these phenomena may be attributed to the following explanations:

According to the available research reports and our observations, absolute or relative hypoxia stimulates increased autocrine MIF in the heart of broiler chickens with ascites, which, in turn, activates AMPK by phosphorylation (Figure 5). Activated AMPK further affects multiple enzymes in the myocardial energy metabolism through substrate phosphorylation, especially by regulating hypoxic myocardial energy metabolism through the following two pathways: In the first pathway, the activated AMPK directly augments glycolysis by phosphorylating and activating an important signaling enzyme in the glycolytic pathway PFK2 [37]. Additionally, PFK1 is the most important rate-limiting enzyme in the glycolytic pathway that catalyzes fructose-6-phosphate (F-6-P) to fructose-1,6-bisphosphate (F-1,6-BP), while PFK2 catalyzes F-6-P to fructose-2,6-bisphosphate (F-2,6-BP), a glycolytic stimulator, which is also the strongest allosteric activator of PFK1 [37]. Thus, PFK2 is also considered a potential activator of PFK1. The glycolytic pathway was regulated by PFK2 by affecting F-2,6-BP levels. Overall, we hypothesized that the cardiac energy metabolism in ascitic broilers may promote glycolysis via the MIF-AMPK-PFK2-PFK1 pathway (Figure 5), which is consistent with the results of Benigni (2000) [38]. The second pathway included the activated AMPK phosphorylating and inhibiting ACC, eventually inhibiting the synthesis of malonyl-CoA. This, in turn, alleviated the inhibition of CPT-1 transporting fatty acyl-CoA to the mitochondrion, eventually promoting fatty acid oxidation [39,40]. Hence, these results suggested that the cardiac energy metabolism in ascitic broilers may promote fatty acid metabolism via the MIF-AMPK-ACC-CPT-1 pathway (Figure 5), which is consistent with the findings of Stanley (1997) [41].

Pyruvate produced by glycolysis reaches two main destinations: first, it produces lactate in the presence of LDH; second, it enters the mitochondrion and produces acetyl-CoA, under the action of the pyruvate dehydrogenase complex (PDHC), which enters the TCA cycle and continues to be oxidized for energy supply. Therefore, we measured the expression levels of both LDHB and PDHE1 to determine the destinations of pyruvate generated by glycolysis.

LDH in animals is a tetramer, which is composed of two kinds of subunits, A and B, which can be assembled into four combinations. LDH exhibits five isozyme forms [42]. Among them, LDHB is mainly present in the myocardium, which catalyzes lactate and generates pyruvate. Our results showed a decreased expression level of LDHB, suggesting the enhanced conversion of pyruvate to lactate by glycolysis in the right hearts of ascitic broilers.

PDHC is a multi-enzyme complex, which catalyzes the oxidative decarboxylation of pyruvate to acetyl-CoA. PDHE1 is one of the components of this complex and is the main site of regulation of PDHC activity, which directly affects the production of acetyl-CoA by pyruvate [43]. The activity of PDHE1 is inhibited by its catalytic product acetyl-CoA through a feedback mechanism. The results showed a decreased expression level of PDHE1, suggesting a relatively high concentration of acetyl-CoA. In vivo biochemical reactions demonstrated that the negative feedback regulation of enzyme activity by enzymatic catalytic products is an important mechanism for maintaining a relatively stable reaction rate. The activity of PDHC, a key enzyme in the glucose metabolism pathway [44], is regulated by the negative feedback of its product, which is important for maintaining the relative stability of glucose metabolism. Our results suggested that the high concentration of acetyl-CoA in the myocardia of ascitic broilers resulted in feedback that inhibited the activity of PDHC, which reduced the rate of glucose metabolism. This entire phenomenon is attributed to the fact that, during the complete oxidative metabolism of glucose and fatty acids, acetyl-CoA, an intermediate product of both pathways, eventually enters the TCA cycle for aerobic oxidation. In other words, acetyl-CoA, the intermediate product of energy metabolism in mitochondria, has two main sources, namely, glycolysis and fatty acid oxidation [45]. However, as an energy molecule, the main destination of acetyl-CoA is the TCA cycle for complete oxidation. The TCA cycle is an aerobic catabolic process, and, since ascitic broilers are in a relatively anoxic state, the rate of the TCA cycle is low, indicating the low efficiency of acetyl-CoA entering the TCA cycle. However, glycolysis and fatty acid oxidation occurring before the production of acetyl-CoA require relatively less oxygen to produce more acetyl-CoA. Although the source of acetyl-CoA changes only a little, the destinations are reduced, and more acetyl-CoA is accumulated. This feedback mechanism, in turn, inhibits and reduces the activity of PDHC.

## 5. Conclusions

In summary, our results showed that the myocardial energy supply in ascitic broiler chickens occurs mainly through glycolysis and fatty acid metabolism. Both MIF and AMPK are involved in the process. However, since the oxidation of the TCA cycle remains blunted, it results in insufficient energy metabolism in the right heart, causing a state of functional failure. This finding indicates that cardiac energy metabolism in broiler chickens is altered during the development of the syndrome. These results may provide useful information to elucidate the pathogenesis of broiler ascites syndrome and may also provide a reference for future research on similar diseases in humans and domestic animals.

## Data Availability

Data supporting the results of this study shall, upon appropriate request, be available from the corresponding author.
